# P-420. Epidemiology of Patients with Laboratory-confirmed *Aspergillus* species in a Singaporean Hospital

**DOI:** 10.1093/ofid/ofae631.621

**Published:** 2025-01-29

**Authors:** Shalvi Arora, Aung Myat Oo, May Kyawt Aung, Darius Y W Chan, Mabel Z Q Foo, Yong Yang, Liang En Wee, Jean Xiang Ying Sim, Moi Lin Ling, Indumathi Venkatachalam

**Affiliations:** Singapore General Hospital, Singapore, Not Applicable, Singapore; Singapore General Hospital, Singapore, Not Applicable, Singapore; Singapore General Hospital, Singapore, Not Applicable, Singapore; Singapore General Hospital, Singapore, Not Applicable, Singapore; Singapore General Hospital, Singapore, Not Applicable, Singapore; Singapore General Hospital, Singapore, Not Applicable, Singapore; Singapore General Hospital, Singapore, Not Applicable, Singapore; Singapore General Hospital, Singapore, Not Applicable, Singapore; Singapore General Hospital, Singapore, Not Applicable, Singapore; Singapore General Hospital, Singapore, Not Applicable, Singapore

## Abstract

**Background:**

*Aspergillus* species is an opportunistic mould associated with serious complications, healthcare costs and mortality, especially in immunocompromised patients. WHO has declared *Aspergillus fumigatus* as a “critical pathogen”. This study aims to report clinical epidemiology of patients with *Aspergillus species* in Singapore General Hospital (SGH), an acute-care hospital.

**Table 1:**
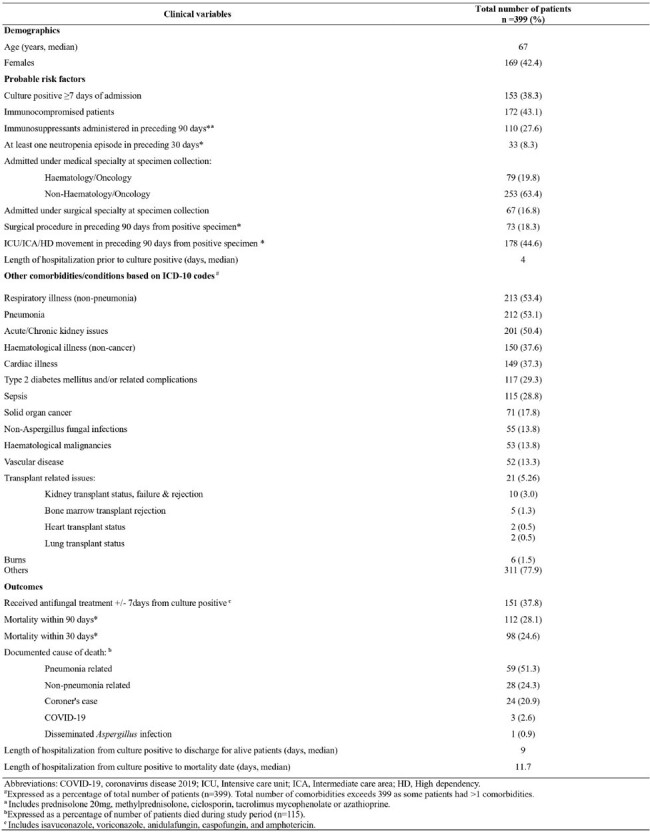
Demographic and clinical characteristics of patients

**Methods:**

Inpatients with laboratory-confirmed *Aspergillus* species were included in this retrospective, observational study between January 2018 and April 2024. For patients with > 1 positive cultures, first positive culture was used to describe clinical characteristics. Comorbidities were identified using ICD-10AM codes. Immunocompromised patients were those under haematology-oncology at time of specimen collection, received immunosuppressants within preceding 90 days and/or were neutropenic (count < 1x10^9^/L) within 30 days preceding specimen collection. Trends were reported as monthly incident rates (IR) per 10,000 patient-days.

**Table 2:**
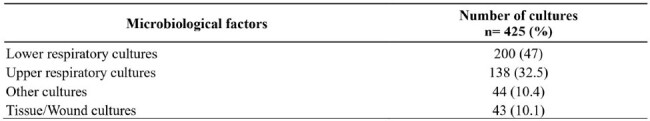
Breakdown of microbiology cultures

**Results:**

The mean IR was 1.2 per 10,000 patient days (n=399). Median patient age was 67 years. 151 of 399 (38%) patients tested positive on a specimen taken ≥ 7 days after admission. 172 (43%) patients were immunocompromised. 213 (53%) patients had non-pneumonia respiratory illnesses, 211 (53%) had pneumonia and 201 (50%) had acute/chronic kidney disease. 79 (20%) patients were admitted under haematology-oncology and 53 (14%) had haematological malignancies. 151 (38%) patients received *Aspergillus* targeted anti-fungal treatment within ±7 days of positive result (surrogate for infections). Crude mortality was 29% (n=115) of whom 98 (85%) patients died within 30 days and 112 (97%) patients died within 90 days of positive specimen. Median duration between specimen collection and mortality was 11.7 days. Pneumonia was the most common cause of death (51%). 187 (47%) patients had positive *Aspergillus* species cultured from lower respiratory specimens. The common species was *Aspergillus fumigatus* complex (45%). No outbreaks were identified during this period.

**Table 3:**
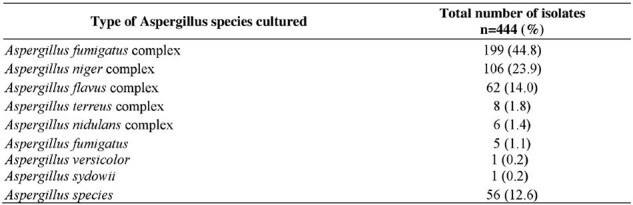
Breakdown by Aspergillus species isolated

**Conclusion:**

*Aspergillus* species causes significant morbidity and mortality in hospitalized patients, including non-immunocompromised. Hence hospital-wide surveillance should be implemented.

**Figure:**
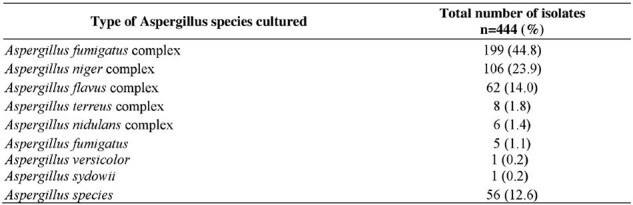

Trend of monthly incidence rates of laboratory-confirmed healthcare-associated Aspergillus patients

**Disclosures:**

**Moi Lin Ling, FRCPA**, Solventum: Honoraria|Solventum: Educational grant for APSIC projects

